# Cost‐effectiveness of a quality improvement bundle for emergency laparotomy

**DOI:** 10.1002/bjs5.62

**Published:** 2018-06-14

**Authors:** C. Ebm, G. Aggarwal, S. Huddart, M. Cecconi, N. Quiney

**Affiliations:** ^1^ Department of Anaesthesia and General Management Wiener Privatklinik (WPK) Vienna Vienna Austria; ^2^ Department of Anaesthesia Royal Surrey County Hospital Guildford UK; ^3^ Department of Intensive Care Medicine St George's Healthcare Trust and St George's University of London London UK

## Abstract

**Background:**

The recent Emergency Laparotomy Pathway Quality Improvement Care (ELPQuiC) study showed that the use of a specific care bundle reduced mortality in patients undergoing emergency laparotomy. However, the costs of implementation of the ELPQuiC bundle remain unknown. The aim of this study was to assess the in‐hospital and societal costs of implementing the ELPQuiC bundle.

**Methods:**

The ELPQuiC study employed a before–after approach using quality improvement methodology. To assess the costs and cost‐effectiveness of the bundle, two models were constructed: a short‐term model to assess in‐hospital costs and a long‐term model (societal decision tree) to evaluate the patient's lifetime costs (in euros).

**Results:**

Using health economic modelling and data collected from the ELPQuiC study, estimated costs for initial implementation of the ELPQuiC bundle were €30 026·11 (range 1794·64–40 784·06) per hospital. In‐hospital costs per patient were estimated at €14 817·24 for standard (non‐care bundle) treatment versus €15 971·24 for the ELPQuiC bundle treatment. Taking a societal perspective, lifetime costs of the patient in the standard group were €23 058·87, compared with €19 102·37 for patients receiving the ELPQuiC bundle. The increased life expectancy of 4 months for patients treated with the ELPQuiC bundle was associated with cost savings of €11 410·38 per quality‐adjusted life‐year saved.

**Conclusion:**

Implementation of the ELPQuiC bundle is associated with lower mortality and higher in‐hospital costs but reduced societal costs.

## Introduction

Emergency laparotomy has been shown to have a high mortality rate^1^ ^2^. Several studies^3^, ^4^, ^5^ have now demonstrated that significant reductions in mortality are possible using an evidence‐based care bundle approach, which streamlines processes, standardizes care, improves quality and ultimately reduces mortality and morbidity.

One study, the Emergency Laparotomy Pathway Quality Improvement Care (ELPQuiC) bundle, was conducted in four general district hospitals in England and used a six‐point pathway^3^. The six points were: prompt assessment using an early warning score; early use of antibiotics when sepsis was diagnosed; operation within 6 h of a decision to operate; use of intraoperative goal‐directed therapy; ICU admission for all patients after surgery; and consultant surgeon and anaesthetist involvement throughout the pathway.

For the ELPQuiC study, the quality improvement methodology used was based on the ‘model for improvement’^6^. This included ongoing feedback and evaluation of data and performance throughout the study to assist implementation of the care bundle. The publication of this project followed the recommendations of the Standards for Quality Improvement Reporting Excellence (SQUIRE) statement^7^.

Despite growing evidence showing improved outcomes linked to clinical pathways^3^, ^4^, ^5^, concerns about additional costs often hamper widespread and rapid adoption^8^. Implementation costs, costs due to increased ICU admissions, and the presence of consultant surgeons and consultant anaesthetists may increase the hospital expenditure costs for survivors and lead to increased hospital costs. This problem is further aggravated by the current UK National Health Service (NHS) reimbursement scheme, which not only incurs a significant shortfall for emergency laparotomy^9^ ^10^ but also, owing to a lack of long‐term outcomes and cost‐effectiveness studies, often ignores potential long‐term cost savings for society.

Under such circumstances, cost‐effectiveness studies can be used to assess costs and benefits. By combining quantitative and qualitative data, the most efficient and cost‐effective solution for a clinical problem can be evaluated^11^, ^12^, ^13^. Those outcomes help decision‐makers (NHS funding) to derive evidence‐based decisions on long‐term costs savings and, importantly, individual and societal wellbeing^14^, ^15^, ^16^.

The primary aim of this study was to compare the in‐hospital costs of a patient receiving bundled care *versus* standard care, with a secondary aim of modelling the cost‐effectiveness of the bundle *versus* standard treatment over the patient's lifetime. In addition, the existing NHS reimbursement scheme for emergency laparotomies was assessed to identify whether current funding is commensurate for individual hospitals and society in general.

## Methods

This was a *post hoc* analysis of the ELPQuiC project, using data from the original ELPQuiC publication^3^. The analysis was conducted in accordance with the Consolidated Health Economic Evaluation Reporting Standards (CHEERS) guidelines^17^.

To assess the cost‐effectiveness of the ELPQuiC pathway, two decision trees were designed. A hospital management perspective was adopted for the short‐term model. This looked at costs associated with implementation of the pathway during the in‐hospital time of the patient. The main outcomes for this model were overall costs per patient of implementing the ELPQuiC bundle compared with standard care.

To construct a decision tree from the hospital's perspective, further data from the ELPQuiC database were obtained that had not been published previously. The Postoperative Morbidity Score (POMS)^18^ was collected for each patient in the ELPQuiC study on days 3 and 7 after surgery and costed using NHS reference costs^19^. Input data were derived from the study and included effectiveness data (30‐day and in‐hospital mortality and morbidity) and cost data (resource utilization during the study period, implementation and in‐hospital costs).

To assess long‐term benefit, a societal perspective was undertaken. This looked at estimated life expectancy of all survivors and the likely cost to gain one additional year of living in perfect health (1 quality‐adjusted life‐year, QALY). To determine the QALY value, the remaining life expectancy for each surviving patient was calculated^20^, and a utility value was assigned to various stages of the disease process^21^, ^22^, ^23^. For example, if an individual has a life expectancy of 7 years, and the QALY value is 0·66 for the first year after operation, 0·71 for the second and third years, and 0·75 for the last 4 years, the adjusted life expectancy is 5·08 years (1*0·66 + 2*0·71 + 4*0·75). Finally, life expectancy was adjusted to account for increased risk of death following an ICU episode and complications after surgery^24^, ^25^, ^26^, ^27^.

Long‐term effects were calculated by extrapolating the results using external evidence on utility and risk of death after survival in the ICU^21^, ^22^, ^23^, ^24^, ^25^, ^26^. A predictive modelling technique was then applied. Follow‐up data from the study were combined with published research data to estimate long‐term costs and outcomes.

### Costs

Costs comprised implementation costs, in‐hospital costs, and postdischarge/lifetime costs. International prices, if applicable, were converted to euros using 2016 exchange rates (conversion day 20 March 2016); £1 = €1·2828. A discount rate of 3·5 per cent was applied in accordance with NHS guidelines^28^.

Costs for implementing the bundled pathway, including planning, training and supervision, were calculated. Additional equipment purchased, such as monitoring devices, and pharmaceutical costs, were also estimated^29^, ^30^, ^31^, ^32^.

In‐hospital costs were calculated based on length of stay and the various levels of care received by each individual patient over time, multiplied by NHS reference costs^19^. Ward, ICU (levels 1–3), and postanaesthetic care unit periods and reoperations were accounted for and calculated as a cost per day. Additional human resource time included anaesthetic and surgical consultants caring for the patient during surgery^32^, as well as ICU nurses performing goal‐directed therapy^31^.

For patients who died in hospital, actual care received and length of stay for each individual up to the date of death were determined. Indirect costs (such as loss of income) were accounted for by using a flat rate.

### Cost‐effectiveness analysis

Two separate decision trees were modelled, from a hospital and a societal perspective, and results were displayed as cost per in‐hospital patient and cost per QALY gained. All costs and outcomes were based on a bootstrap analysis with 10 000 replications and presented as means with 95 per cent confidence intervals. A cost‐effectiveness acceptability curve was generated to display the degree of acceptance relative to the costs generated by the intervention.

### Sensitivity analysis

A deterministic and probabilistic sensitivity analysis was conducted to account for assumptions in the input parameter and to determine the robustness of the model. In the first step, discount rate, length of stay, number of patients per site, life expectancy following high‐risk surgery, and utility values were examined using a one‐way sensitivity analysis. The key drivers (most ‘sensitive’ parameters) were then subjected to two‐ and three‐way analyses to test the combined parameter uncertainty. For a better understanding, key drivers were then displayed graphically in a tornado diagram.

## Results

**Figure 1:**
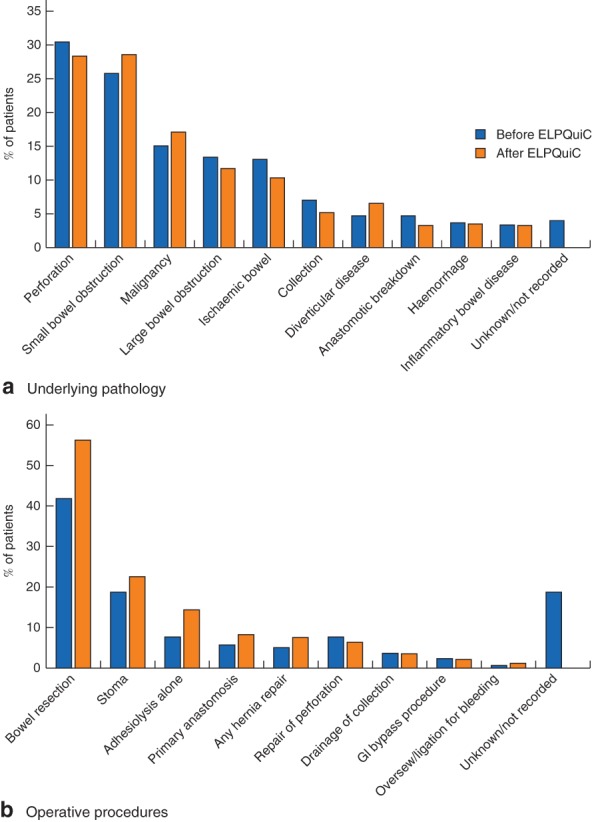
**a** Underlying pathology and **b** operative procedures performed (as a proportion of all patients) before (299 patients) and after (427 patients) implementation of the Emergency Laparotomy Pathway Quality Improvement Care (ELPQuiC) bundle. GI, gastrointestinal

A total of 299 patients underwent emergency laparotomy before implementation of the bundle in this study, compared with 427 consecutive patients after implementation. The underlying pathology and surgery performed, derived from the ELPQuiC database, are shown in *Fig*. 1. Overall, the indications for surgery were similar in the two groups of patients. Bowel resection was performed more commonly in the postimplementation group, whereas more patients had ‘unknown’ surgical procedures in the preimplementation group. The overall risk‐adjusted mortality rate decreased from 15·6 to 9·6 per cent (risk ratio 0·61, 95 per cent c.i. 0·45 to 0·84; *P* = 0·002). The overall crude 30‐day mortality rate decreased from 14·0 (95 per cent c.i. 10·1 to 18·0) per cent in the baseline group to 10·5 (7·6 to 13·5) per cent following implementation of the care bundle. Mean in‐hospital stay was 11 (i.q.r. 7–23) days before implementation and 11 (6–21) days after implementation.

### Short‐term model: cost‐effectiveness

Model input data are shown in *Table* 1. Mean total spending for implementing the bundle at each hospital was €30 026·11 (range 1794·64–40 784·06), driven largely by the purchase of cardiac output devices, as well as teaching and supervision by a consultant trainer. Assuming an average equipment amortization of 5 years and an eligible patient population of 50 patients per site per year, implementation was associated with a mean cost of €600·48 (range 17·96–2039·27) per patient.

**Table 1:** Model input data

	Unit	Value*	Reference
Implementation			
Training, supervision and administration			
Salary			
Nurses	€	29 553·15 (19 242–38 484)	31
Consultants	€	100 856·05 (96 517·87–130 140·06)	32
Registrars	€	88 383·00 (66 705·60–110 060·39)	32
Time commitment (per week)			
Nurses (3)	h	6·0 (3–24)	Trial data
Consultants (2)	h	12·0 (4–16)	Trial data
Registrars (3)	h	6·0 (2–24)	Trial data
Cardiac output monitors	€	24 493·78	11
Bundle			
Tazocin^®^	€	19·50	30
Operating room (rate/min)	€	20·52	3
Incremental operating room time	min	5·17	Trial data
Cost of goal‐directed fluid therapy/patient	€	129·56 (128·28–615·74)	11
Consultant anaesthetist (incremental time commitment)	%	17	Trial data
Consultant surgeon (incremental time commitment)	%	18	Trial data
Variable costs			
Cost of ward bed	€	256·56	19
Costs of ICU bed (levels 1–5)	€	(1113·47–2238·49)	19
Long‐term costs			
Costs of long‐term complication	€	406·65 (304·02–507·99)	33
Utility†			
After 12 months		0·66	23
After a complication		0·51	23

* Values in parentheses are ranges.

† Utility is a ratio (0–1): 0 is the utility of death; 1 is the utility of full health. £1 = €1·2828 (exchange rate 20 March 2016). Tazocin^®^: Pfizer, Tadworth, UK.

Incremental mean costs related to the ELPQuiC bundle (additional presence of consultant in theatre, pharmaceuticals and goal‐directed therapy) were €361·11 (range 323·78–878·97) per patient (*Table* 2).

**Table 2:** Resource use and outcome of patients undergoing emergency laparotomy with standard care compared with ELPQuiC care‐bundled care

	Standard care (non‐ELPQuiC)	ELPQuiC bundle care
30‐day mortality (%)	14·0	11·5
Length of stay		
ICU (days)		
Level 1	7·3	0·2
Level 2	1·6	2·0
Level 3	2·6	1·7
Ward (days)	8·7	14·6
Costs (€)		
ICU	15 174·37	6338·44
Ward	3140·04	5261·79
Complications	8242·12	10 137·71
In‐hospital death	15 326·77	10 404·66
Implementation		30 026·11
ELPQuiC bundle		361·11 (323·78–878·97)
Life expectancy (years)	6·70	7·21
Life expectancy (quality‐adjusted years)	4·53	4·88
Cost‐effectiveness (short term)		
Costs (€)	14 817·24	15 971·24
Effectiveness/utility*	0·7	0·9
ICER (€ per patient)	–	12 066·00
Net monetary benefit (€)	9781·73	14 262·43
Cost‐effectiveness (societal)		
Costs (€)	23 058·87	19 102·37
Effectiveness/utility*	4·5	5·87
ICER (€ per patient)	–	−11 410·38
Net monetary benefit (€)	28 185·54	33 529·20

Values are mean (range).

* Utility is a ratio (0–1): 0 is the utility of death; 1 is the utility of full health. £1 = €1·2828 (exchange rate 20 March 2016). ELPQuiC, Emergency Laparotomy Pathway Quality Improvement Care; ICER, incremental cost‐effectiveness ratio.

For the short‐term (hospital‐based) model, mean total costs per patient associated with the bundle were €15 971·24, compared with costs for standard treatment of €14 817·24. Adjusting for decreased survival in the standard treatment group (0·7 life‐years *versus* 0·9 life‐years in the ELPQuiC group) revealed an incremental cost‐effectiveness ratio (ICER) of €12 066·66 per patient (*Table* 2).

Using POMS data and the short‐term hospital model decision tree, it was identified that the occurrence of postoperative complications gave rise to additional costs. Patients who developed complications incurred costs of €10 367·72–25 031·66, compared with €3039·72–18 163·93 in patients who had no complications. The highest expenses were found in the group that received standard care, required ICU admission, and developed a complication (mean cost €25 031·66). The main drivers were complication costs and length of stay in the ICU.

Costs for patients who died were €15 326·77 per patient in the standard group and €10 404·66 per patient in the ELPQuiC group.

**Figure 2:**
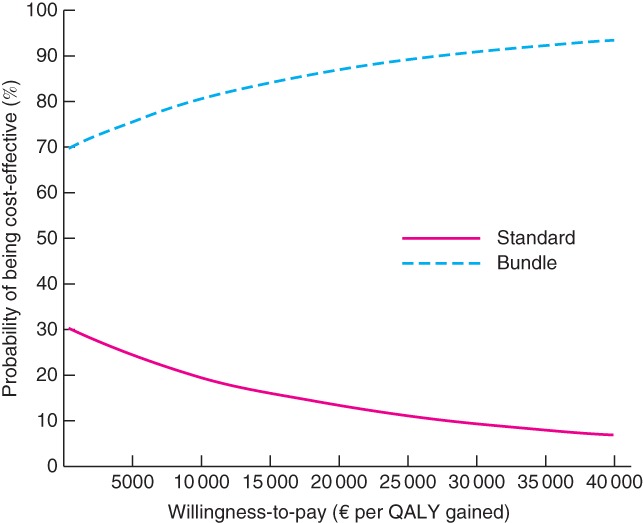
Cost‐effectiveness acceptability curve for standard and bundle treatments, illustrating the uncertainty surrounding the estimate of the cost‐effectiveness analysis

To address the uncertainty around the estimates of mean costs and outcomes, a cost‐effectiveness acceptability curve was generated (*Fig*. 2). This can be used to display the probability of being cost‐effective at various thresholds (the decision‐maker's willingness‐to‐pay). A range of cost‐effectiveness thresholds was plotted on the horizontal axis against the probability that the intervention would be cost‐effective at that threshold on the vertical axis. Assuming the currently accepted NHS threshold of £30 000 (€38 484)/QALY gained, the intervention was 90·7 per cent cost‐effective. Even when a willingness‐to‐pay of £0 (€0)/QALY gained was assumed, the probability of the intervention being considered cost‐effective was still 70 per cent.

### Societal model: cost‐effectiveness

Mean(s.d.) long‐term costs associated with the ELPQuiC bundle were €19 102·37(14 661·12) with a mean(s.d.) quality‐adjusted life expectancy of 4·88(0·28) years, compared with €23 058·87(10 227·76) and 4·53(0·25) years respectively for standard care. The mean discounted and quality‐adjusted life expectancy in the intervention group increased by 0·34 life‐years, or 4 months. The calculated ICER was negative with € − 11 484·40/QALY, which is a result of positive (more effective) clinical outcome in the nominator and negative (lower) costs of the pathway in the denominator (Table 2).

**Figure 3:**
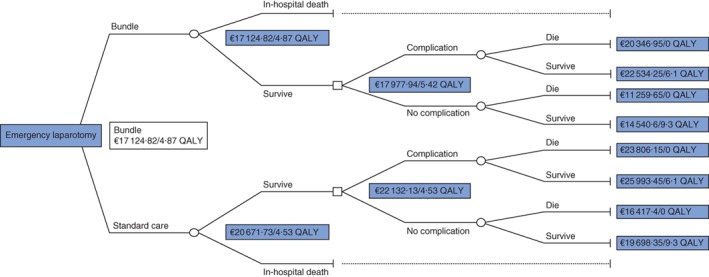
Societal model. At the initiation node, a patient enters either the standard or the Emergency Laparotomy Pathway Quality Improvement Care (ELPQuiC) bundle branch. Throughout the hospital stay, the individual may die, develop complications or remain alive without complication, and accrues costs. Mean outcomes are calculated by summing the probability of reaching each state with the input parameter (costs and utility). Costs include the main expenses (in‐hospital, pharmaceutical, follow‐up costs) accrued during the observation period. QALY, quality‐adjusted life‐years gained by receiving standard or bundle care

Detailed outcomes of the long‐term (societal perspective) decision tree are shown in Fig. 3.

## Discussion

Using health economic modelling and data collected from the ELPQuiC study, estimated costs for initial implementation of the ELPQuiC bundle were estimated at €30 026·11 (range 1794·64–40 784·06) per hospital. In‐hospital costs per patient were estimated at €14 817·24 for standard (non‐care bundle) treatment *versus* €15 971·24 for the ELPQuiC bundle. Improved survival for patients receiving the ELPQuiC bundle was associated with increased hospital costs of €12 066·66 per additional life saved. The long‐term model showed that the intervention was both more effective (increased mean survival of 4 months) and led to lower costs to society, with savings of €3956·50 per survivor. The ICER met the commonly accepted NHS threshold of £30 000 (€38 484)/QALY. Sensitivity analysis confirmed the robustness of the model results.

The ELPQuiC pathway not only improves clinical care but also reduces mortality and is cost‐effective in the long term. Therefore, it should be recommended as a quality improvement programme to policy and financial decision‐makers.

In this evaluation, historical mean hospital costs of €14 817·24–15 971·24 per patient undergoing emergency laparotomy were calculated. This is similar to the average expenses reported by Shapter and colleagues^9^ of £13 000 (€16 676·40) and by Murray and co‐workers^34^ of median £9282 (range 6222–14 400) (€11 906·95 (7981·58–18 472·32)). Although there are variations in NHS reimbursement rates (payment by results tariffs) of approximately £4000–7000 (€5131·20–8979·60) per emergency case^35^, these results confirm that significant NHS funding deficits exist for each patient (funding shortfall of £4550–6450 (€5836·74–8274·06)). Such funding gaps may have detrimental effects on the willingness of providers to invest in quality improvement programmes. This ongoing deficit highlights the need for cost‐effectiveness modelling to demonstrate the benefits of investment in high‐cost clinical areas^36^.

Some of the calculated costs associated with bundle implementation may be excessive. In particular, the incremental costs of introducing goal‐directed fluid therapy or the additional resources required to continue this therapy in the ICU may be inflated. If this is the case, the ELPQuiC bundle may be cheaper than described, thereby lowering the cost per additional life saved.

The use of bundles of care is now widespread. Many observers associate improved outcomes with reduced costs. Although this may be aspirational, the results may not be forthcoming owing to difficulty in assessing costs. The use of evidence from care bundles, such as ELPQuiC, will allow commissioners to identify areas of spending where maximum return, in terms of lives saved or economic benefit to society overall, can be most effective.

This is the first study comparing the economic impact of an emergency laparotomy pathway with standard care. Thus, although these findings indicate a positive effect on outcome and costs, caution should be applied. There are several limitations that need to be considered. First, no long‐term study data on quality of life and cost data were available. The ELPQuiC study reported both in‐hospital and 30‐day mortality; longer‐term outcomes were not available. Suggestions for using longer outcome data were published after completion of the ELPQuiC project^37^.

The model used in this study combined trial input data with international evidence on long‐term data. This may introduce bias, as utility of life and costing may vary across countries and healthcare systems. As this approach was the only possibility to model long‐term outcomes, those parameters were tested in a sensitivity analysis. When varying the parameter, and assuming maximal costs and minimal utility for long‐term survival, the model still revealed robust data and the pathway remained the cost‐effective strategy.

Second, the life expectancy of survivors was adjusted to account for the hazard ratio (HR) of survivors following high‐risk surgery and presenting with postoperative complications. In a 15‐year follow‐up study, Rhodes *et al*.^25^ found an HR of 1·93; thus a similar value was used for the present study population. In the sensitivity analysis, the ICER remained negative when varying the life expectancy, indicating that even worst life expectancy was associated with the care bundle remaining the dominant treatment. Length of patient stay is the primary determinant of hospital costs. It may be that with a longer implementation time ELPQuiC could reduce length of hospital stay and therefore overall hospital costs.

The ELPQuiC project was a quality improvement project not a blinded RCT. ELPQuiC was designed not to discover new evidence, but to assess the effect of consistent delivery of evidence‐based care for patients undergoing emergency laparotomy. Criticism of this project may be forthcoming, in particular the small number of participating sites, the non‐randomization of patients, the lack of a power estimate, the use of a ‘before and after’ approach, and the fact that a formal, pre‐experimental protocol is not available. However, quality improvement work generally describes improvement activities in a well‐defined service or problem. Quality improvement projects act as ‘proof of concept’ rather than to define new knowledge as outputs of RCTs. Following on from ELPQuiC, a larger‐scale quality improvement collaborative approach is now being undertaken^37^.

The main strength of this study was costing patient care from both a hospital and a societal perspective. Improvement in mortality has been shown in this study to increase costs (but within commonly accepted thresholds) for hospitals, but to decrease overall costs to society. Further work is warranted to continue to deliver improved mortality, but also to reduce length of stay for patients undergoing emergency laparotomy.
